# Validity and reliability analysis of the Korean version of the Partners in Health Scale for middle-aged women

**DOI:** 10.1371/journal.pone.0362383

**Published:** 2026-11-13

**Authors:** Haein Lee, Junghee Kim

**Affiliations:** 1 College of Nursing, Dankook University, Cheonan, Republic of Korea; 2 Department of Nursing, Seoil University, Seoul, Republic of Korea; Universita degli Studi Europea di Roma, ITALY

## Abstract

**Purpose:**

Middle-aged women experience persistent and multifaceted health-related changes during the menopausal transition that require ongoing self-management. However, validated instruments for assessing self-management in this population are limited. Therefore, this study aimed to evaluate the validity and reliability of the Korean version of the Partners in Health Scale for middle-aged women (K-PIH-MW).

**Methods:**

A secondary analysis was conducted using data from 312 community-dwelling Korean women aged 45–64 years. Content validity was assessed by six experts. Construct validity was examined through confirmatory factor analysis (CFA), including factor-level convergent and discriminant validity. Convergent construct validity was also evaluated using theoretically related external measures. Internal consistency reliability was assessed using Cronbach’s α, Spearman–Brown coefficients, and McDonald’s ω, as appropriate.

**Results:**

The K-PIH-MW demonstrated good content validity (scale-level content validity index = .96). CFA supported the original four-factor structure, with acceptable model fit (comparative fit index = .97, Tucker-Lewis index = .96, root mean square error of approximation = .08, standardized root mean square residual = .03). Factor-level convergent and discriminant validity among the four domains were supported. Convergent construct validity was supported by significant positive correlations with self-care agency (r = .63, *p* < .001), physical health-related quality of life (r = .31, *p* < .001), and mental health-related quality of life (r = .40, *p* < .001). The overall internal consistency was high (Cronbach’s α = .92), with satisfactory domain-level reliability based on Spearman–Brown coefficients and McDonald’s ω.

**Conclusion:**

These findings support the validity and internal consistency of the K-PIH-MW as a multidimensional measure of self-management among middle-aged women. The instrument may be useful for assessing self-management across multiple domains and identifying areas that may require additional support in clinical and community settings.

## Introduction

Middle age is a period marked by diverse physical and psychological changes in women, including the menopausal transition, which is characterized by a gradual decline in ovarian function accompanied by changes in hormone levels [1]. Menopause is defined retrospectively after 12 consecutive months of amenorrhea following the final menstrual period [2]. Across the menopausal transition, women may experience a range of symptoms, including vasomotor symptoms, musculoskeletal pain, urogenital symptoms, mood changes, and sleep disturbances, along with an increased risk of chronic diseases (e.g., cardiovascular disease, osteoporosis, and diabetes) [1,3,4]. These health challenges may persist for years, affecting physical and psychosocial well-being as well as long-term health [1,3,5]. These persistent and multifaceted health challenges highlight the importance of active health management throughout midlife [6].

Self-management refers to individuals’ active participation in learning and practicing the skills needed to manage their chronic health conditions [7]. Lorig and Holman [8] described self-management as the ability to manage medical, emotional, and role-related consequences of long-term health conditions. Although the menopausal transition is a physiological process rather than a chronic condition, persistent symptoms and health concerns during this period may require ongoing self-management [6]. In middle-aged women, self-management may involve managing bothersome symptoms and promoting long-term health through lifestyle modification, symptom monitoring, health- and treatment-related decision-making, and partnerships with healthcare providers [6,9,10]. Evidence from systematic reviews indicates that higher levels of self-management among middle-aged women are associated with reduced menopausal symptoms, lower levels of anxiety and depression, improved health-related quality of life (HRQoL), and a reduced risk of chronic health problems such as metabolic syndrome and cardiovascular disease [11,12].

Given the importance of self-management for these health outcomes, its accurate assessment is essential for identifying self-management support needs and developing tailored interventions [13,14]. However, available measures for middle-aged women have generally focused on selected aspects of self-management, such as the management of specific symptoms (e.g., fatigue and urinary incontinence) [15,16] or specific self-care practices and health behaviors [17,18], rather than capturing the broader multidimensional processes of self-management. Although a previous study of middle-aged women used the Appraisal of Self-Care Agency Scale (ASAS) to assess self-management [19], the ASAS primarily assesses self-care agency, focusing on individuals’ underlying capacity for self-care [20]. In contrast, self-management encompasses the broader processes of managing ongoing health needs, including symptom management, health-related decision-making, and partnership with healthcare professionals [21]. Therefore, an instrument that captures the broader, multidimensional processes of self-management is needed for middle-aged women.

The Partners in Health (PIH) scale was developed as a generic measure of self-management [21]. Although the PIH has primarily been applied in populations with chronic conditions, it assesses four domains of self-management: knowledge, symptom management, coping, and partnership with healthcare providers [21–23]. These domains reflect aspects of ongoing health management relevant to middle-aged women, who may experience diverse and persistent health changes requiring continuous adaptation and management [24]. Therefore, the PIH scale may provide a suitable multidimensional framework for assessing self-management among middle-aged women.

In Korea, the existing Korean version of the PIH (PIH-K) has been validated among individuals diagnosed with chronic conditions [25]. However, several items contain wording that assumes the presence of a diagnosed disease, current treatment, or disease-related symptoms. Such assumptions may not be consistently applicable to middle-aged women, who may experience menopausal symptoms or other health concerns without having a diagnosed chronic condition or receiving ongoing treatment. Accordingly, the PIH-K was contextually adapted to improve its applicability to middle-aged women while retaining its multidimensional self-management framework. Given these modifications, psychometric evaluation of the adapted scale in the target population was warranted [26]. Therefore, this study evaluated the validity and reliability of the Korean version of the Partners in Health Scale for Middle-aged Women (K-PIH-MW). We hypothesized that the K-PIH-MW would retain the original four-factor structure and show evidence of convergent construct validity through positive associations with self-care agency and physical and mental HRQoL.

## Methods

### Study design

This study employed a secondary analysis to evaluate the validity and reliability of the K-PIH-MW. Data were accessed for research purposes on 15 October 2025. The original survey did not collect personally identifiable information, and the authors had no access to information that could identify individual participants either during or after data collection. Data were originally collected by the authors’ research team to investigate the association between health literacy and self-management among middle-aged women. Participant recruitment and data collection were conducted from 5 February to 31 May 2024. The original data collection was approved by the Institutional Review Board (approval no. CUIRB-2023-0057). The present secondary analysis was reviewed by the Institutional Review Board and was granted an exemption (approval no. DKU 2025-10-025).

### Participants

Participants were female adults aged 45–64 years, residing in community settings in South Korea. Participants were recruited through convenience sampling from public health centers and community organizations located in Daegu, Ulsan, Gyeonggi Province, and Seoul. The selected age range reflects prior evidence indicating that the average age at natural menopause among Korean women is approximately 50 years, with menopausal symptoms typically emerging approximately five years before menopause. Eligible participants were native Korean speakers who could communicate and complete the self-administered questionnaire and had no cognitive, visual, or auditory impairments. Women with incurable or terminal illnesses and healthcare professionals were excluded. After obtaining permission from the participating institutions, a total of 317 women completed the questionnaires. After excluding five respondents who did not meet the age criteria, 312 women were included in the final analysis. Based on the commonly recommended ratio of at least 10 participants per item for scale validation [27], the sample size in this study was sufficient.

### K-PIH-MW

The PIH scale, originally developed by Battersby et al. [21], is a self-report instrument designed to assess self-management in individuals with chronic conditions. It was initially introduced as an 11-item tool [21] and was later refined into a 12-item version [23,28] that has been widely translated and validated across various cultures and chronic conditions [29–32]. The revised version consists of 12 items rated on a 9-point Likert scale ranging from 0 (not at all) to 8 (very much) [23,28]. The total scores range from 0 to 96, with higher scores indicating higher levels of self-management. Jeon et al. [25] translated and culturally adapted the PIH into Korean (PIH-K) following the WHO guidelines through forward translation, expert panel review, back-translation, and reconciliation. In their validation study, the PIH-K was administered to individuals diagnosed with chronic illnesses who had been taking physician-prescribed medications for at least three months. The PIH-K consists of 12 items across four domains: Knowledge (2 items), Partnership in treatment (4 items), Recognition and management of symptoms (2 items), and Coping (4 items). The PIH-K showed Cronbach’s α coefficients of 0.68 for Knowledge, 0.77 for Partnership in treatment, 0.61 for Recognition and management of symptoms, and 0.83 for Coping [25].

In the present study, the PIH-K items were reviewed for their contextual relevance to middle-aged women. Based on this review, Items 2, 3, 4, and 7 were selected for contextual adaptation, while the remaining eight items were retained without modification. The selected items were adapted while preserving the multidimensional self-management framework of the original PIH. Previous literature [1,4,33] was used to identify health concerns relevant to middle-aged women and to inform the contextual wording of the adapted items.

First, Item 2 was revised from “treatment” to “management or treatment” to encompass health management beyond clinical treatment. Similarly, in the Korean wording of Item 7, the phrase referring to symptoms “related to my disease” was changed to “related to my health condition.” In addition, illness-specific indicators (e.g., blood sugar levels, shortness of breath, and nausea) were replaced with health concerns more relevant to middle-aged women, such as weight gain, pain, sleep problems, and depression. Second, Items 3 and 4 were reformulated to assess participants’ ability to perform the behaviors described in the items, as the original wording presupposed a current treatment context and may not have been applicable to women who were not receiving treatment. Item 3 retained its focus on following treatment recommendations, while Item 4 retained its focus on communication and shared decision-making with healthcare professionals. These modifications were intended to improve applicability to women with varying health and treatment situations while preserving the self-management behaviors represented by the original items. The original and modified wording of the four adapted items are provided in English in S1 Table in S1 File.

This initial version of the K-PIH-MW was then evaluated through content validity assessment by six experts with relevant clinical and research experience, including nursing professors and gynecological nurses with at least 5 years of teaching or clinical experience. It demonstrated good content validity (scale-level content validity index [CVI] = .96, with item-level CVI values ≥ .83). As part of the same content validity assessment, the six experts also provided qualitative comments on the relevance and wording of the adapted items. Based on these comments, Item 7 was further refined from “weight gain” to “weight change” to better reflect variability in weight-related changes among middle-aged women. This refinement was incorporated into the finalized instrument used for data collection and all subsequent psychometric analyses.

### Measures

#### Self-care agency.

The Appraisal of Self-Care Agency Scale–Revised (ASAS-R) is an instrument designed to assess general self-care agency. The original ASAS was developed by Evers et al. [34], and its revised version was subsequently validated by Sousa et al. [20]. The Korean version of the ASAS-R consists of 15 items across three domains: having power for self-care, developing power for self-care, and lacking power for self-care [35]. Items are rated on a 5-point Likert scale ranging from 1 (strongly disagree) to 5 (strongly agree), with total scores ranging from 15 to 75 [35]. Higher scores indicate greater self-care agency. In this study, the Korean version of the ASAS-R demonstrated good internal consistency (Cronbach’s α = .87).

#### Health-related quality of life.

We employed the RAND 36-Item Short Form Health Survey (SF-36) to capture the participants’ HRQoL. This questionnaire, originally developed by Ware and Sherbourne [36], was translated into Korean and shown to have acceptable psychometric properties in prior research conducted by Koh et al. [37]. The SF-36 assesses multiple dimensions of perceived health through 36 items that collectively represent eight domains (i.e., physical functioning, role limitations due to physical health, pain, general health, vitality, social functioning, role limitations due to emotional problems, and emotional well-being). For analytical purposes, domain scores were summarized into two composite indices: Physical Component Summary (PCS) and Mental Component Summary (MCS), reflecting overall physical and mental health status [36].

In accordance with established scoring conventions [38], responses were standardized using norm-based scoring procedures referenced to the U.S. general population, yielding T-scores with a mean of 50 and a standard deviation (SD) of 10. Higher scores indicate better HRQoL. In this study, the SF-36 demonstrated acceptable internal consistency, with Cronbach’s α coefficients across subscales ranging from .59 to .82.

#### Participant characteristics.

Participants’ characteristics included sociodemographic and health-related variables. Sociodemographic variables included age, education level, family co-residence, and employment status. Education level was categorized as high school graduation or below and college graduation or above. Family co-residence was classified as living alone or living with family members, and employment status was categorized as employed or unemployed.

Health-related variables included menopausal status, chronic disease, sleep quality, and current hormone therapy use. Menopausal status was categorized as pre/perimenopausal or postmenopausal. Postmenopause was defined as the absence of menstruation for at least 12 consecutive months [2], and all other participants were categorized as pre/perimenopausal. Chronic disease was assessed by self-report of any physician-diagnosed chronic disease. Sleep quality was assessed using a single-item measure of overall sleep quality during the past month, rated on a five-point Likert scale ranging from 1 (very poor) to 5 (very good). Responses were dichotomized into good (very good or good) and poor (fair, poor, or very poor). Current hormone therapy use was identified based on self-reported use of hormonal preparations, including estrogen, progesterone, or oral contraceptives.

### Statistical analysis

Statistical analyses were performed using Stata version 17.0 [39]. Descriptive statistics, including means, SDs, frequencies, and percentages, were used to summarize participants’ characteristics. Item analysis for the K-PIH-MW was performed by examining item means, SDs, and corrected item–total correlations (CITC), with CITC values of ≥ .30 considered indicative of adequate item discrimination [40]. In addition, data normality was confirmed, as all item distributions met the criteria of absolute skewness < 3 and kurtosis < 10 [41].

The content and construct validity of the K-PIH-MW were evaluated. Content validity was assessed using the CVI based on expert evaluation, with item-level CVI values ≥ .83 and a scale-level CVI ≥ .90 considered acceptable [42]. Construct validity was examined through CFA using the maximum likelihood estimation method. Model fit was evaluated using multiple fit indices, including χ² with degrees of freedom and *p*-value, comparative fit index (CFI ≥ .90), Tucker–Lewis index (TLI ≥ .90), root mean square error of approximation (RMSEA ≤ .08) with its 90% confidence interval (CI), and standardized root mean square residual (SRMR ≤ .08) [43]. As an additional analysis, measurement invariance across pre/perimenopausal and postmenopausal groups was examined among participants with complete data for the multigroup CFA. Configural, metric, and scalar invariance were sequentially tested. Measurement invariance was evaluated based on changes in CFI, RMSEA, and SRMR according to the criteria proposed by Chen [44]. For the present sample size and unequal group sizes, noninvariance was indicated by a decrease in CFI of ≥ .005 accompanied by either an increase in RMSEA of ≥ .010 or an increase in SRMR of ≥ .025 for metric invariance and ≥ .005 for scalar invariance [44].

At the factor level, convergent and discriminant validity were evaluated as aspects of the internal measurement structure. Convergent validity was assessed using the average variance extracted (AVE), with values ≥ .50 considered acceptable [43]. Construct reliability (CR) was also calculated, with values ≥ .70 considered acceptable [43]. Based on the Fornell–Larcker criterion [45], discriminant validity was considered acceptable when the square root of the AVE for each factor exceeded the inter-factor correlations. These analyses were used to evaluate convergence within and empirical distinction between the latent factors, respectively. In addition, convergent construct validity with theoretically related external measures was examined using Pearson’s correlation coefficients of the K-PIH-MW with the ASAS-R and SF-36 measures. Internal consistency reliability was assessed using Cronbach’s α for the total scale and subscales. In addition, McDonald’s ω was calculated for the four-item subscales [43], whereas the Spearman–Brown coefficient was used for the two-item subscales [46]. Cronbach’s α values between .70 and .95 were considered indicative of adequate internal consistency [47].

## Results

### Participant characteristics

Participants’ mean age was 51.3 years, and 71.7% had a college degree or higher. Most participants (97.1%) were living with family members, and 80.8% were employed. More than half of the participants (58.4%) were pre/perimenopausal and 63.7% reported no diagnosed chronic diseases. A large proportion (71.4%) reported poor sleep quality, and the majority (95.1%) were not receiving current hormone therapy (Table 1).

**Table 1:** Participant characteristics (*N* = 312).

Variable	Frequency (%) or Mean ± SD	
Age (year)^a^	51.3 ± 4.5	
Education level		
High school graduation or below	87	(28.3)
College graduation or above	220	(71.7)
Family co-residence		
No	9	(2.9)
Yes	301	(97.1)
Employment status		
Employed	249	(80.8)
Unemployed	59	(19.2)
Menopausal status		
Pre/perimenopause	177	(58.4)
Postmenopause	126	(41.6)
Chronic disease		
No	177	(63.7)
Yes	101	(36.3)
Sleep quality		
Good	88	(28.6)
Not good	220	(71.4)
Current hormone therapy use		
No	293	(95.1)
Yes	15	(4.9)

^a^Mean ± SD.

SD = standard deviation.

### Item analysis

Item analysis indicated that the mean scores of the 12 items of the K-PIH-MW ranged from 5.09 to 7.04. The CITC coefficients ranged from .50 to .76, exceeding the acceptable threshold of .30 [40] (Table 2). Additionally, skewness values (ranging from −1.43 to 0.19) and kurtosis values (ranging from 1.79 to 4.20) were within the predefined acceptable ranges [41].

**Table 2:** Item analysis and internal consistency of the K-PIH-MW (*N* = 312).

Item	Mean ± SD	Corrected item-total correlation	Cronbach’s α if item deleted
1. Overall, what I know about my health condition(s) is	5.22 ± 1.32	.69	.92
2. Overall, what I know about my management or treatment of my health condition(s) is	5.15 ± 1.44	.71	.91
3. I can take medications or carry out the treatments asked by my doctor or health worker	6.72 ± 1.48	.63	.92
4. I can share(discuss) in decisions made about my health condition(s) with my doctor or health worker	6.60 ± 1.45	.66	.92
5. I am able to deal with health professionals to get the services I need that fit with my culture, values and beliefs	6.47 ± 1.55	.57	.92
6. I attend appointments as asked by my doctor or health worker	7.04 ± 1.37	.50	.92
7. I keep track of my symptoms and early warning signs related to my health condition(s) (e.g., weight change, pain, sleep problems, and depressive symptoms)	5.73 ± 1.55	.74	.91
8. I take action when my early warning signs and symptoms get worse	5.82 ± 1.54	.76	.91
9. I manage the effect of my health condition(s) on my physical activity (e.g., walking, household tasks)	5.36 ± 1.84	.71	.92
10. I manage the effect of my health condition(s) on how I feel (e.g., my emotions and spiritual wellbeing)	5.33 ± 1.75	.75	.91
11. I manage the effect of my health condition(s) on my social life (e.g., social interactions)	5.39 ± 1.72	.72	.91
12. Overall, I manage to live a healthy life (e.g., no smoking, moderate alcohol, healthy food, regular physical activity, manage stress)	5.09 ± 1.89	.66	.92

K-PIH-MW = Korean version of the Partners in Health Scale for Middle-aged Women.

### Reliability

The K-PIH-MW demonstrated high internal consistency, with an overall Cronbach’s α of .92. The deletion of individual items did not improve internal consistency (Table 2). Cronbach’s α coefficients for the four domains were .94 for knowledge, .88 for partnership in treatment, .84 for recognition and management of symptoms, and .92 for coping, indicating acceptable internal consistency across the domains. For the two-item domains, the Spearman–Brown coefficients were .94 for knowledge and .84 for recognition and management of symptoms. For the four-item domains, McDonald’s ω coefficients were .88 for partnership in treatment and .92 for coping.

### Construct validity

CFA was conducted to examine the construct validity of the K-PIH-MW. The results indicated that the original four-factor structure was retained in this population (i.e., knowledge, partnership in treatment, recognition and management of symptoms, and coping). Standardized factor loadings ranged from .66 to .96, and all loadings were statistically significant, indicating that the items adequately represented their respective constructs. Although the chi-square test was statistically significant (χ^2^(48) = 133.1, *p* < .001), this test is sensitive to sample size [43]. Considering the other fit indices (CFI = .97, TLI = .96, RMSEA = .08, 90% CI [.06, .09], SRMR = .03), the proposed four-factor model demonstrated an acceptable fit (Fig 1).

**Fig 1:**
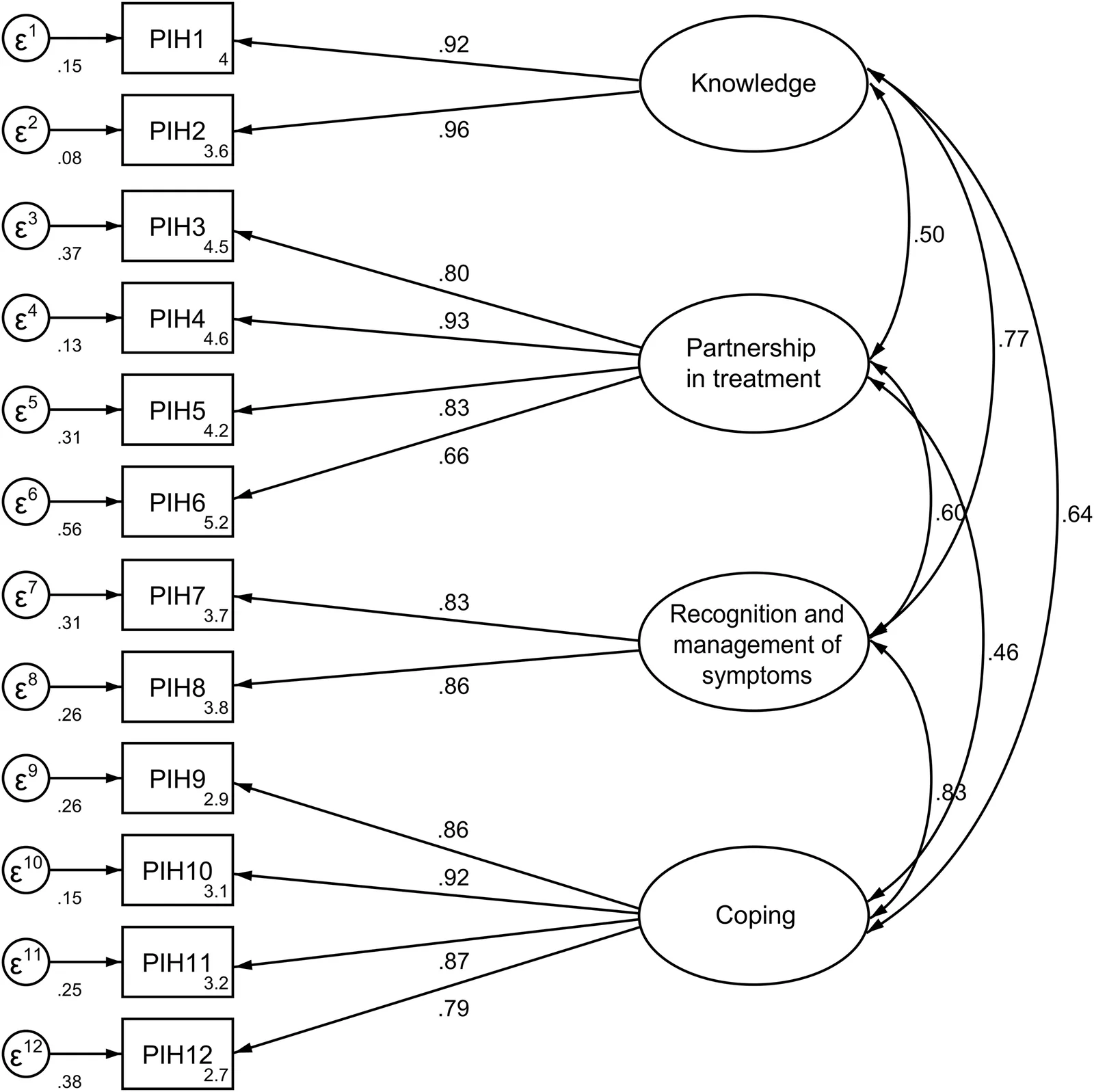
Confirmatory factor analysis of the K-PIH-MW. K-PIH-MW = Korean version of the Partners in Health Scale for Middle-aged Women; Overall model fit: χ^2^(48) = 133.1, *p* < .001, CFI = .97, TLI = .96, RMSEA = .08, 90% CI [.06, .09], SRMR = .03.

At the factor level, AVE values supported convergence within the respective factors (range = .66–.88), while CR values were acceptable (range = .83–.94). The Fornell–Larcker criterion was also met, supporting empirical distinction among the four domains (Table 3).

**Table 3:** Factor-level measurement properties of the K-PIH-MW (*N* = 312).

Factor	Mean	SD	AVE	CR	Knowledge	Partnership in treatment	Recognition and management of symptoms	Coping
Knowledge	10.38	2.69	.88	.94	(.94)			
Partnership in treatment	26.84	5.01	.66	.88	.50^*^	(.81)		
Recognition and management of symptoms	11.56	2.86	.71	.83	.77^*^	.60^*^	(.84)	
Coping	21.14	6.45	.74	.92	.64^*^	.46^*^	.83^*^	(.92)

K-PIH-MW = Korean version of the Partners in Health Scale for Middle-aged Women; SD = standard deviation; AVE = average variance extracted; CR = construct reliability.

Diagonal elements in parentheses represent the square roots of AVE.

* *p* < .001.

As an additional analysis, measurement invariance across pre/perimenopausal and postmenopausal groups was examined using multigroup CFA among participants with complete data for the analysis. Configural, metric, and scalar invariance were supported, with changes in CFI, RMSEA, and SRMR remaining within the recommended criteria (S2 Table in S1 File).

### Convergent construct validity

The convergent construct validity of the K-PIH-MW was evaluated using Pearson’s correlation analysis with the ASAS-R and SF-36 scores. The K-PIH-MW score demonstrated a significant positive correlation with the ASAS-R score (r = .63, *p* < .001). The K-PIH-MW scores were significantly and positively correlated with both the PCS (r = .31, *p* < .001) and the MCS (r = .40, *p* < .001) of the SF-36, supporting the convergent construct validity of the scale (Table 4).

**Table 4:** Convergent construct validity of the K-PIH-MW with the ASAS-R and SF-36 (*N* = 312).

Variable	K-PIH-MW	ASAS-R	SF-36		Mean ± SD	Min	Max
			PCS	MCS			
K-PIH-MW	—				69.93 ± 13.93	29	96
ASAS-R	.63^**^	—			50.58 ± 7.65	30	72
SF-36							
PCS	.31^**^	.30^**^	—		46.95 ± 6.74	21.56	62.37
MCS	.40^**^	.36^**^	.18^*^	—	46.64 ± 8.17	18.48	63.77

K-PIH-MW = Korean version of the Partners in Health Scale for Middle-aged Women; ASAS-R = Appraisal of Self-Care Agency Scale–Revised; PCS = Physical Component Summary; MCS = Mental Component Summary; SD = standard deviation.

* *p* < .01, ** *p* < .001.

## Discussion

This study provided evidence supporting the validity and internal consistency of the K-PIH-MW as a measure of self-management among middle-aged women. By refining the PIH-K to reflect the health management context of middle-aged women, the present study extends the applicability of the original instrument beyond diagnosis-specific clinical populations. These findings support the four-factor structure, factor-level measurement properties, convergent construct validity, and internal consistency of the instrument. Retention of the original four-factor structure supports the multidimensional structure of self-management as assessed by the K-PIH-MW.

The contextual refinements introduced in the K-PIH-MW improve its applicability to middle-aged women in two ways. First, wording in the knowledge and recognition and management of symptoms domains was broadened to better accommodate health experiences that may not be framed as a diagnosed illness. This adjustment allows middle-aged women to reflect on a wider range of midlife health experiences, including fluctuating symptoms such as weight changes, pain, and sleep problems, which are common during midlife and may extend beyond a disease-specific framing. Second, two items in the partnership in treatment domain were reformulated to assess participants’ ability to perform the behaviors described in the items, as the original wording presupposed a current treatment context. This modification improved their applicability to women who were not receiving treatment while retaining the behaviors represented by the original items [48]. These findings support the applicability of the four-factor PIH structure to middle-aged women in a broader health context.

Factor-level analyses generally supported the distinctiveness of the four domains, although the strength of the inter-factor correlations varied. The strongest correlation was observed between recognition and management of symptoms and coping, which may reflect their conceptual relatedness, as recognizing and responding to symptoms is closely linked to broader coping and health-management behaviors in daily life. Despite this strong association, the two domains remained empirically distinguishable, supporting the structural coherence of the adapted four-factor model. These findings support the structural coherence of the adapted four-factor model, which retained the original four-factor structure of the PIH [25,28,49]. The additional measurement invariance analysis further supported a comparable four-factor measurement structure across pre/perimenopausal and postmenopausal groups. In particular, the retention of the partnership domain suggests that the multidimensional self-management framework of the K-PIH-MW captures not only individual knowledge and symptom-related management but also a collaborative dimension involving communication and health-related decision-making with healthcare professionals. This interpretation is consistent with menopause care literature highlighting collaborative decision-making and patient–provider partnership as important components of menopause-related health management [9,10].

The convergent construct validity findings indicate that the K-PIH-MW is meaningfully related to self-care agency, while capturing a distinct dimension of self-management. Its significant positive association with the ASAS-R suggests that self-management in middle-aged women is closely related to self-care agency, which has been described as a prerequisite for effective self-care [20,50,51]. Simultaneously, the moderate magnitude of this association indicates that the two instruments are related but not redundant, reflecting their different conceptual emphases [51]. Self-care agency refers to an individual’s capability to recognize self-care needs, evaluate relevant resources, and determine self-care actions [50]. In contrast, the K-PIH-MW captures a broader, multidimensional process of self-management relevant to middle-aged women [8,21,51], including understanding of health management or treatment, shared decision-making with healthcare providers, and monitoring and coping with symptoms commonly experienced during midlife.

The associations with HRQoL provided further evidence for the convergent construct validity of the K-PIH-MW. The K-PIH-MW was significantly associated with both mental and physical HRQoL, with a stronger association observed for mental HRQoL, consistent with previous studies [52,53]. Self-management involves active problem solving, symptom management, and partnership with health professionals, which may be more closely related to psychosocial functioning than to physical functioning [53]. In contrast, the comparatively weaker association with physical HRQoL may reflect the distinction between self-management and physical health status. Physical HRQoL may also be influenced by clinical burden, such as multimorbidity [54], which could further attenuate its association with self-management. Therefore, although the observed associations support convergent construct validity, the relatively modest association with physical HRQoL should be interpreted cautiously in terms of its clinical significance.

The internal consistency of the K-PIH-MW was satisfactory, with an overall Cronbach’s α of .92. This finding is consistent with the satisfactory internal consistency previously reported for the original revised PIH (α = .82) and the PIH-K (α = .86) [25,28]. Domain-level estimates, including Cronbach’s α, Spearman–Brown coefficients, and McDonald’s ω, also indicated satisfactory internal consistency. These findings suggest that the contextual refinements did not compromise the reliability of the adapted scale.

This study has several strengths. First, it validated a multidimensional instrument for assessing self-management in middle-aged women, for whom few such validated instruments are available. Second, this study provides psychometric evidence regarding the factor structure, factor-level convergent and discriminant validity, convergent construct validity, and internal consistency. However, this study also has some limitations that must be acknowledged. First, the use of convenience sampling may limit the generalizability of the findings, given the relatively high proportions of participants with higher education and employment and those without chronic conditions. Second, although the adapted items underwent expert content validation, cognitive interviews or formal face-validity assessment with middle-aged women were not conducted, limiting the evaluation of their comprehensibility and perceived relevance from the perspective of the target population.

### Practical implications

Considering the importance of self-management during the menopausal transition, the K-PIH-MW may be useful for assessing multidimensional self-management among middle-aged women in clinical and community settings. By assessing self-management beyond selected health behaviors or individual menopausal symptoms, the instrument enables healthcare professionals to consider how women manage midlife health challenges, cope with their broader impacts, and engage in collaborative partnerships with healthcare providers [8]. Such assessment may help identify areas of self-management that require additional support and inform the prioritization of targeted health care support. With further evidence regarding responsiveness and meaningful score interpretation, the K-PIH-MW may also have potential as an outcome measure for self-management education and support programs.

## Conclusion

This study supports the validity and internal consistency of the K-PIH-MW as a measure of self-management among middle-aged women. The K-PIH-MW retained the original four-factor structure of the PIH-K while providing a contextually adapted measure for assessing multidimensional self-management within the broader health context of midlife, beyond diagnosis-specific clinical situations. The instrument may be useful in research and practice for assessing self-management and identifying areas that may require additional support among middle-aged women.

Further research is needed in several areas. First, additional validation should be conducted with more diverse groups of middle-aged women to examine the broader applicability of the K-PIH-MW across different sociodemographic and health-related contexts. Second, longitudinal studies are needed to examine the instrument’s stability over time (e.g., test-retest reliability) and responsiveness to change following self-management interventions. Third, future research should examine whether the K-PIH-MW functions similarly across subgroups of middle-aged women, such as those differing in symptom burden or chronic disease experience. Additional studies are also warranted to establish known-groups validity and clinically meaningful score interpretation.

## Supporting information

S1 File Supplementary Tables S1-S2. This file contains two supplementary tables. S1 Table presents the original PIH-K and modified K-PIH-MW wording for the adapted items, and S2 Table presents the measurement invariance results across pre/perimenopausal and postmenopausal women.
(DOCX)
