# Supplementary material for: Validity and reliability analysis of the Korean version of the Partners in Health Scale for middle-aged women
Source: PLoS One. 2026 Nov 13;21(11):e0362383. doi: 10.1371/journal.pone.0362383 (PMC13766227; doi:10.1371/journal.pone.0362383)
Supplement: Supplementary file 1 [file pone.0362383.s001.docx]

S1 Table.

Original PIH-K and Modified K-PIH-MW Wording for the Adapted Items

| Items | Original PIH-K | K-PIH-MW |
| --- | --- | --- |
| 2 | Overall, what I know about the treatment, including medications of my health condition(s) is | Overall, what I know about the management or treatment of my health condition(s) is |
| 3 | I take medications or carry out the treatments asked by my doctor/health worker | I can take medications or carry out the treatments asked by my doctor or health worker. |
| 4 | I share in decisions made about my health condition(s) with my doctor or health worker | I can share in decisions made about my health condition(s) with my doctor or health worker. |
| 7 | I keep track of my symptoms and early warning signs (e.g. blood sugar levels, peak flow, weight, shortness of breath, pain, sleep problems, mood). | I keep track of symptoms and early warning signs related to my health condition(s) (e.g., weight change, pain, sleep problems, and depressive symptoms). |

PIH-K = Korean version of the Partners in Health Scale; K-PIH-MW = Korean version of the Partners in Health Scale for Middle-Aged Women. The original wording presented in this table is from the PIH-K translated and validated by Jeon et al. [1], based on the original Partners in Health Scale [2-4]. Items 2, 3, 4, and 7 were contextually adapted for the K-PIH-MW; the remaining eight items (Items 1, 5, 6, and 8–12) were unchanged from the PIH-K. The Partners in Health Scale, including its adapted materials, is copyright of Flinders University.

S2 Table.

| Model | χ² | df | CFI | TLI | RMSEA | SRMR | ΔCFI | ΔRMSEA | ΔSRMR |
| --- | --- | --- | --- | --- | --- | --- | --- | --- | --- |
| Configural invariance | 190.303 | 96 | .967 | .954 | .081 | .036 | – | – | – |
| Metric invariance | 196.075 | 104 | .967 | .959 | .077 | .037 | .000 | -.004 | .001 |
| Scalar invariance | 202.599 | 112 | .968 | .962 | .074 | .037 | .001 | -.003 | .000 |

Measurement Invariance of the K-PIH-MW Across Pre/perimenopausal and Postmenopausal Women	(*n* = 298)

PIH = Partners in Health scale; CFI = comparative fit index; TLI = Tucker–Lewis index; RMSEA = root mean square error of approximation; SRMR = standardized root mean square residual. ΔCFI, ΔRMSEA, and ΔSRMR represent the **change in fit indices relative to the** previous model.

**References**

1.	Jeon M-K, Ahn J-W, Park Y-H, Lee M-K. Validity and Reliability of the Korean Version of the Partners In Health Scale (PIH-K). J Korean Crit Care Nurs. 2019;12(2):1-12. https://doi.org/10.34250/jkccn.2019.12.2.1.

2.	Petkov J, Harvey P, Battersby M. The internal consistency and construct validity of the partners in health scale: validation of a patient rated chronic condition self-management measure. Quality of Life Research. 2010;19(7):1079-85. https://doi.org/10.1007/s11136-010-9661-1.

3.	Smith D, Harvey P, Lawn S, Harris M, Battersby M. Measuring chronic condition self-management in an Australian community: factor structure of the revised Partners in Health (PIH) scale. Quality of Life Research. 2017;26(1):149-59. https://doi.org/10.1007/s11136-016-1368-5.

4.	Battersby MW, Ask A, Reece MM, Markwick MJ, Collins JP. The Partners in Health scale: The development and psychometric properties of a generic assessment scale for chronic condition self-management. Australian Journal of Primary Health. 2003;9(2-3):41-52.
